# Comments by the Academic Editors to Responses and Replies Concerning Mroczek et al.’s “Evaluation of Quality of Life of Those Living near a Wind Farm“: *Int. J. Environ. Res. Public Health* 2015, *12*, 6066–6083

**DOI:** 10.3390/ijerph14030272

**Published:** 2017-03-08

**Authors:** Peter Lercher, Paul B. Tchounwou

**Affiliations:** 1 Medical University Innsbruck, Christoph Probst Platz 1, Innrain 52, A-6020 Innsbruck, Austria; 2 Molecular Toxicology Research Laboratory, Jackson State University, 1400 Lynch Street, Box 18750, Jackson, MI 39217, USA; paul.b.tchounwou@jsums.edu

We are aware of the current divide in the scientific assessment of the potential health effects of wind turbine installations. Therefore, a transparent, scientific, and open debate is necessary, and it is our duty as editors and reviewers to take such a critique seriously.

There were six major points of critique expressed by Shepherd [1] regarding the paper of Mroczek et al. [2] in this journal. The editors received another comment; however, the person did not want to have the comment published. This comment is, however, covered by Shepherd’s comments.

We reassessed the paper [2] and the critique [1] and came to the following conclusions.

## 1. Duplicate Publication

The authors of this general critique are not fully satisfied. They responded quite correctly; the current article is a more “in-depth analysis” of the data [3].

In addition, the major conclusion in the abstract is slightly different from the older 2012 paper [4], “*Within all scales, the quality of life was assessed highest by residents of areas located closest to wind farms, and the lowest by those living more than 1500 m from wind farms*” was changed to a more differentiated description: “*The lowest overall QoL and general health scores were noted among residents of places where windfarm developments were either at the stage of planning or under construction*”.

However, this conclusion is selectively taken as a message, and, given the wide variety of the additional—partly contradictory—results from the new analysis, a more modest conclusion would have been more appropriate.

## 2. Conflicts of Interest

There is no doubt that the first author would have been obliged by a variety of international ethic rules to report about her husband’s (Jaroslaw Mroczek) involvement as the Polish Wind Energy Association (PWEA) President in the Polish windfarm community, and as a member of the Top Management team in the EPA Sp. z o.o. around the time the study was conducted. Jaroslaw Mroczek is currently (time of publication in 2015) President of the EPA Sp. z o.o.

It is a requirement to disclose such relationships—especially in cases where no direct money from the windfarm community was involved in the project.

The International Committee of Medical Journal Editors (ICMJE) Form for Disclosure of Potential Conflicts of Interest states clearly in Section 5 [5]: “*Are there other relationships or activities that readers could perceive to have influenced, or that give the appearance of potentially influencing, what you wrote in the submitted work?*”

## 3. Scientific Critique on Methods: Presentation of SF-36 Summary Scores

Shepherd [1]:

Omission Three: The means for the SF-36 Scale were not reported for the groups of interest. This makes it very difficult to determine whether the differences between the groups (i.e., effect sizes) were substantial or not. This is important when one has large sample sizes such as those reported in the Mroczek et al. study (n = 1277), as very small and clinically insignificant differences may in fact reach statistical significance. In fact, the means reported in their Table Four (p. 6074) are totally irrelevant to their research objectives ...

Shepherd is right on this point. In order to judge the obtained results—instead of presenting the overall means—the relevant tables should show the means by the respective group (e.g., by distance to wind farm, by respondents who did not accept the development, by wind-farm development in the planning stage, etc.)

Furthermore, as “*Age is the sociodemographic factor that had the greatest effect on QoL in the multiple regression model*”, stratification by age would have been important (note also the wide age range of this study).

Furthermore, general and specific reservations against the use of summary scores of the SF-36 exist in the literature (e.g., Simon et al. 1998 [6], Wilson et al. 2000 [7], and Hawthorne et al. 2007 [8]).

In response to such reservations, Ware & Kosinski (2001) [9] responded: “*we again repeat our 7-year-old recommendation that results based on summary measures should be thoroughly compared with the SF-36 profile before drawing conclusions*”.

Another point made by Shepherd merits further investigation: “*while the authors laud the ‘availability of normative data’ (p. 6070), they do not compare their data to these*”.

In their response, the authors replied: “*As normative we regarded data which come from international studies”* and quote the *User Manual for the SF-36v2 Health Survey*.

Under Point 7 (below), there are several reasons mentioned that suggest that the survey population may depart strongly from typical populations and that a comparison with data from international studies may not be appropriate (especially with those from the USA).

Therefore, the point made by Shepherd has good rationale, as these substantial differences in the health status of the surveyed population in the Mroczek et al. study (which are not discussed in the paper) would have suggested following the approach Shepherd indicated. Moreover, Hawthorne et al. (2007) [8] mentioned that the used population norms are important to account for local factors and avoid wrong conclusions.

## 4. Scientific Critique on Included Literature

According to Shepherd [1], Omission One: “*In support of their statement that ‘No scientific evidence has been found so far in favor of the influence of turbines (in particular, of their noise) on health’ (p. 6067) the authors cite two reviews*“ [10,11]. Shepherd regarded these two reviews [10,11] as “*out-dated and widely derided*” and “*also limited” and asks why “[a] more recent and more competently undertaken review from the University of Oxford*” [12] has not been included.

The response by Mroczek et al. [3] was not correct: “*We are familiar with the article of Onakpoya et al. [of] 2015, but it concerns the influence of noise on sleep disorders, which was not a subject of our study*”.

In fact, the review included studies on annoyance and visual impact on health and quality of life and followed the STROBE guidelines [13] for the assessment of study quality.

In addition, it should be mentioned that the summary paper on the annoyance response to wind turbine noise curves from four surveys by Janssen et al. [14] is clearly missing and in contrast to the “*no scientific evidence statement*”.

## 5. Other Missing Points: A Clear Discussion of the Limitations of This Study

- No noise assessment at all was provided.
- No continuous distance measure was used in the analyses—in spite of the fact that the study was a door to door survey, where addresses were known.
- The study was of cross-sectional design—but the interpretation was done in terms of “development stage“ (as if of longitudinal design).

## 6. Other Scientific Remarks

### 6.1. Statistical Issue

The regression model presented in Table 5 [2] used a wide variety of “independent” variables (diseases, symptoms, risky behaviours, etc.), which are known to have high inter-correlations and may be prone to collinearity. However, there was no mention of how this potential problem was treated.

### 6.2. Scientific Critique on Statements in the Results Section

*Irritation (OR = 1.49), anxiety (OR = 0.66), anger (OR = 0.87), and nervousness (OR = 0.80) occurred more frequently in respondents who lived closer to the development than in those living about 2 km from such development. People living close to wind-farm construction sites feel nervous (W = 32.56, p = 0.0001) and angry (W = 46.01, p = 0.0001)*.

[2]

The content of these two sentences is not scientifically sound: First, from the single point estimate of the odds ratios, the statistical significance of this estimate cannot be judged without including confidence intervals.

Second, the signs of the odds ratios are on both sides of 1; however, the interpretation, given in the paper, is the same: it “*occurred more frequently in respondents*”. This is obviously a wrong conclusion.

Third, these results are in strong conflict with the SF-36 results in the discussion and conclusions.

### 6.3. Scientific Critique on Statements in the Discussion and Conclusions

There were several statements in this paper that were not unequivocally founded by the obtained results. “*The available results imply that wind turbines, when located at a proper distance, do not exert any negative effects on human health*” [2].

“*The presence of wind farms near residential areas has no negative influence on the QoL of residents*” [2]. This statement is an inappropriate generalization of the study results to the universe.

The cross-sectional design prohibits such a conclusion—especially when the study population may be quite different and the acoustic features of this windfarm are not at all characterized in this study.

Furthermore, the discrepancy in the results of the SF-36 scores and the symptom analysis should have led the authors to be very cautious in deriving any conclusions beyond this study with this highly differing study population (see Point 7). See COPE guidelines [15]—*12.1. Errors, inaccurate or misleading statements must be corrected promptly and with due prominence*—for reference.

## 7. What Is Different in This Study from Others (and Should Have Been Discussed)

### 7.1. Sociodemographic Factors

A few socio-demographic features are unusual in the sample and should have been sensibly discussed in comparison with other studies—as these factors strongly influence the morbidity prevalence of the sample.

- High unemployment proportion.
- Low proportion of higher education (thus adjustment for education is not working).
- Age range up to 94 (women) and 85 (men).

### 7.2. Health Related Factors

- A very high proportion of people with diseases was described in this study; afflictions included hypertension (26.62%, 340), rheumatism (14.17%, 181), coronary heart disease (12.84%, 164), and diabetes (11.82%, 151). e.g., Fabian et al. in 2005 [16] reported the local prevalence of diabetes in Szczecin (Poland) as 3.56% in age 3–95 years (mean 65.4 ± 13.6 years)
- From international data bases you get the following picture:
  1. The prevalence of diabetes in the study is even higher than expected. The OECD (2013) [17] estimated the prevalence of diabetes for Poland at 9.2%.
  2. Prevalence of hypertension is within the higher range in Europe.
  3. Coronary heart disease (CHD) prevalence is higher than that in other parts of Europe, except for the eastern countries

Overall, there is a higher burden of illness in this survey sample compared with other studies of this kind.

### 7.3. Proportion of Survey Residents Who May Profit from the Windfarm Installation

Different from other studies, a high proportion of survey residents may profit economically from windfarm installations in this study:

- residents who derived real economic benefits by leasing their land (8.65%; *n* = 110)
- residents who expected to be able to lease their land at the planning stage (21.78%; *n* = 277)

A comparison with a similar question from Table 3 in the study of Bakker et al. in 2012 [18] showed that the proportion of residents who may profit somehow from the windfarm installation was only 99/685 = 14.4% in the sample of Bakker et al.

## 8. Other Concerns

### Incomplete Compliance with Reviewers Suggestions

#### Reviewer No. 2 Irritation vs. Annoyance

“*For consistency with the referenced literature, the term irritation should be replaced with the term annoyance throughout the paper*”.

Answer by Mroczek [2]: “*Changed as suggested*”—Editorial comment: There is still irritation in the text and no explanation was given in the paper why annoyance was not used.

“*Where results seem to be contradictory there should also be a discussion about why this might be*”.—Editorial comment: suggestion not considered.

#### Reviewer No. 3

“*There is a need for discussion of study limitations*”.

Editorial comment: suggestion not considered.

## 9. Conclusions

The close involvement of the first author’s husband with the wind turbine industry was not mentioned at the time of submission.

The authors did not follow important advice from the reviewers on central issues (e.g., irritation vs. annoyance, discussion of limitations, and discrepant results).

There are also scientific concerns with the presentation of the SF-36 data.

The conclusions drawn were not appropriate based on the inherent limitations and other peculiarities (see Point 7) of the study population.

**Note from the editor:** This editorial was provided by the Academic Editors in response to the reply provided by Mroczek and co-workers. This is only a part of an extensive discussion. The entire discussion will be also published.
